# Modeling Higher-Order Correlations within Cortical Microcolumns

**DOI:** 10.1371/journal.pcbi.1003684

**Published:** 2014-07-03

**Authors:** Urs Köster, Jascha Sohl-Dickstein, Charles M. Gray, Bruno A. Olshausen

**Affiliations:** 1 Redwood Center for Theoretical Neuroscience, University of California, Berkeley, Berkeley, California, United States of America; 2 Department of Applied Physics, Stanford University and Khan Academy, Palo Alto, California, United States of America; 3 Department of Cell Biology and Neuroscience, Montana State University, Bozeman, Montana, United States of America; Max Planck Institute for Biological Cybernetics and Bernstein Center for Computational Neuroscience, Germany

## Abstract

We statistically characterize the population spiking activity obtained from simultaneous recordings of neurons across all layers of a cortical microcolumn. Three types of models are compared: an Ising model which captures pairwise correlations between units, a Restricted Boltzmann Machine (RBM) which allows for modeling of higher-order correlations, and a semi-Restricted Boltzmann Machine which is a combination of Ising and RBM models. Model parameters were estimated in a fast and efficient manner using minimum probability flow, and log likelihoods were compared using annealed importance sampling. The higher-order models reveal localized activity patterns which reflect the laminar organization of neurons within a cortical column. The higher-order models also outperformed the Ising model in log-likelihood: On populations of 20 cells, the RBM had 10% higher log-likelihood (relative to an independent model) than a pairwise model, increasing to 45% gain in a larger network with 100 spatiotemporal elements, consisting of 10 neurons over 10 time steps. We further removed the need to model stimulus-induced correlations by incorporating a peri-stimulus time histogram term, in which case the higher order models continued to perform best. These results demonstrate the importance of higher-order interactions to describe the structure of correlated activity in cortical networks. Boltzmann Machines with hidden units provide a succinct and effective way to capture these dependencies without increasing the difficulty of model estimation and evaluation.

## Author Summary

Communication between neurons underlies all perception and cognition. Hence, to understand how the brain's sensory systems such as the visual cortex work, we need to model how neurons encode and communicate information about the world. To this end, we simultaneously recorded the activity of many neurons in a cortical column, a fundamental building block of information processing in the brain. This allows us to discover statistical structure in their activity, a first step to uncovering communication pathways and coding principles. To capture the statistical structure of firing patterns, we fit models that assign a probability to each observed pattern. Fitting probability distributions is generally difficult because the model probabilities of all possible states have to sum to one, and enumerating all possible states in a large system is not possible. Making use of recent advances in parameter estimation, we are able to fit models and test the quality of the fit to the data. The resulting model parameters can be interpreted as the effective connectivity between groups of cells, thus revealing patterns of interaction between neurons in a cortical circuit.

## Introduction

Electrophysiology is rapidly moving towards high density recording techniques capable of capturing the simultaneous activity of large populations of neurons. This raises the challenge of understanding how networks encode and process information in ways that go beyond tuning properties or feedforward receptive field models. Modeling the distribution of states in a network provides a way to discover communication patterns between neurons or functional groupings such as cell assemblies which may exhibit a more direct relation to stimulus or behavioral variables.

The Ising model, originally developed in the 1920s to describe magnetic interactions [1], has been used to statistically characterize electrophysiological data, particularly in the retina [2], and more recently for cortical recordings [3], [4]. This model treats spikes from a population of neurons binned in time as binary vectors and captures dependencies between cells with the maximum entropy distribution for pairwise dependencies. This has been shown to provide a good model for small groups of cells in the retina [5], though it is unable to capture dependencies higher than second-order.

In this work, we apply maximum entropy models to neural population recordings from the visual cortex. Cortical networks have proven more challenging to model than the retina: The magnitude and importance of pairwise correlations between cortical cells is controversial [6], [7] and higher-order correlations, i.e. correlations which cannot be captured by a pair-wise maximum entropy model, play a more important role [8]–[10]. One of the challenges with current recording technologies is that we can record simultaneously only a tiny fraction of the cells that make up a cortical circuit. Sparse sampling together with the complexity of the circuit mean that the majority of a cell's input will be from cells outside the recorded population. In adult cat visual cortex, direct synaptic connections have been reported to occur between 11%–30% of nearby pairs of excitatory neurons in layer IV [11], while a larger fraction of cell pairs show “polysynaptic” couplings [12], defined by a broad peak in the cross-correlation between two cells. This type of coupling can be due to common inputs (either from a different cortical area or lateral connections) or a chain of monosynaptic connections. A combination of these is believed to give rise to most of the statistical interactions between recorded pairs of cells. The Ising model, which assumes only pairwise couplings, is well suited to model direct (and symmetric) synaptic coupling, but cannot capture interactions involving more than two cells. We propose a new approach, that addresses both incomplete sampling and common inputs from other cell assemblies, by extending the Ising model with a layer of hidden units or latent variables. The resulting model is a semi-Restricted Boltzmann Machine (sRBM), which combines pairwise connections between visible units with an additional set of connections to hidden units.

Estimating the parameters of energy-based models, to which Ising models and Boltzmann machines belong, is computationally hard because these models cannot be normalized in closed form. For both Ising models and Boltzmann machines with hidden units, the normalization constant is intractable to compute, consisting of a sum over the exponential number of states of the system. This makes exact maximum likelihood estimation impossible for all but the smallest systems and necessitates approximate or computationally expensive estimation methods. In this work, we use Minimum Probability Flow (MPF [13], [14], in the context of neural decoding see [4], [15]) to estimate parameters efficiently without computing the intractable partition function. It provides a straightforward way to estimate the parameters of Ising models and Boltzmann machines for high-dimensional data.

Another challenge in using energy-based models is the evaluation of their likelihood after fitting to the data, which is again made difficult due to the partition function. To compute probabilities and compare the likelihood of different models, annealed importance sampling (AIS) [16] was used to estimate the partition function.

Combining these two methods for model estimation and evaluation, we show that with hidden units, Boltzmann machines can capture the distribution of states in a microcolumn of cat visual cortex significantly better than an Ising model without hidden units. The higher-order structure discovered by the model is spatially organized and specific to cortical layers, indicating that common input or recurrent connectivity within individual layers of a microcolumn are the dominant source of correlations. Applied to spatiotemporal patterns of activity, the model captures temporal structure in addition to dependencies across different cells, allowing us to predict spiking activity based on the history of the network.

## Results

### Modeling laminar population recordings

**Figure 1:**
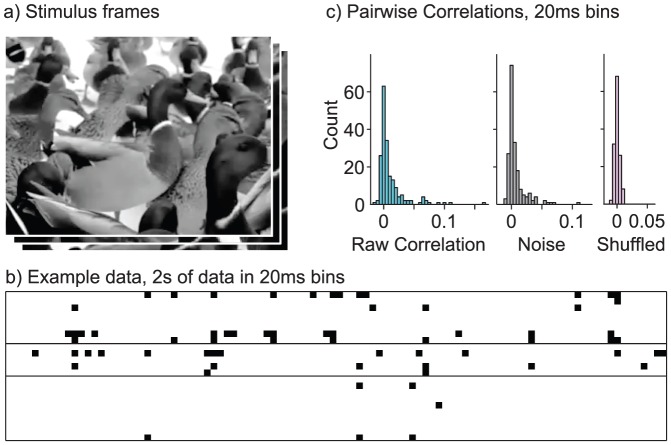
Laminar population recordings in response to natural movies. (**a**) Example of one of the natural movie stimuli, depicting ducks on a lawn, presented full-field at 150 frames per second. (**b**) Example data from 22 cells (session B4), binned in 20 ms windows, 2 s of data. Columns of this matrix form the training data for our algorithm. For the spatiotemporal version of the model, several adjacent columns are concatenated. (**c**) Pairwise correlations in the raw data, and noise correlations computed from 60 repetitions of a 30 s stimulus, binned at 20 ms. Both show small, positive correlations. Shuffling spikes for each of the cells shows that correlations expected due to shared firing rate modulations time-locked to the stimulus are much smaller.

We estimated Ising, RBM and sRBM models for populations of cortical cells simultaneously recorded across all cortical layers in a microcolumn of cat V1 in response to long, continuous natural movies presented at a frame rate of 150 Hz. Code for the model estimation is available for download at http://github.com/ursk/srbm. Fig. 1a) shows an example frame from one of the movies. Model parameters were estimated using MPF with an 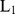 regularization penalty on the model parameters to prevent overfitting. To compute and compare likelihoods, the models were normalized using AIS. Here we present data from two animals, one with 22 single units (B4), another with 36 units (T6), as well as a multiunit recording with 26 units (B4M). Fig. 1b) shows spiking data from session B4 in 20 ms bins, with black squares indicating a spike in a bin. Spatiotemporal datasets were constructed by concatenating spikes from consecutive time bins. Pairs of cells show weak positive correlations, shown in Fig. 1c), and noise correlations computed from 60 repetitions of a 30s stimulus are similarly small and positive. For all recordings, the population was verified to be visually responsive and the majority of cells were orientation selective simple or complex cells. As recordings were performed from a single cortical column, receptive fields shared the same retinotopic location and have similar orientation selectivity, differing mostly in size, spatial frequency and phase selectivity. See [17] for a receptive field analysis performed on the same raw data.

### Pairwise and higher-order models

**Figure 2:**
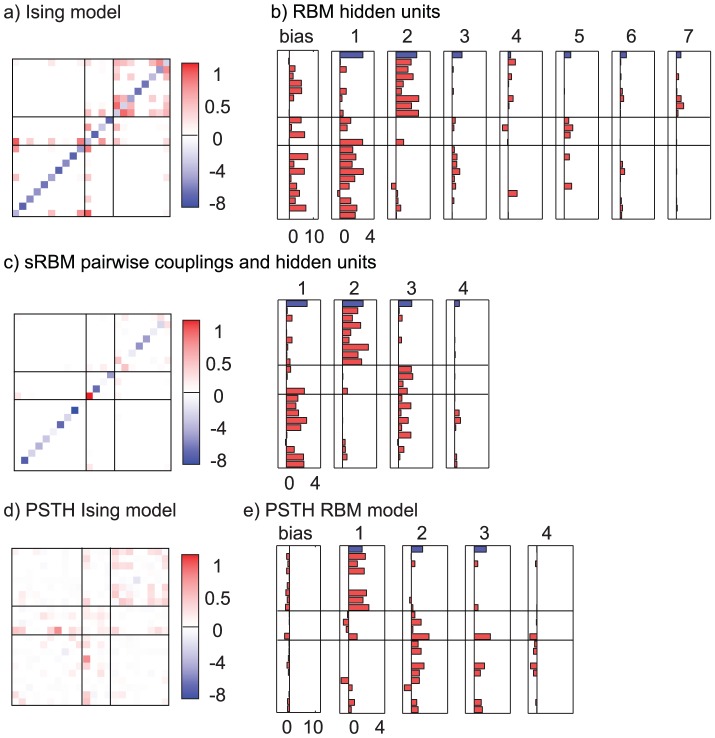
Functional connectivity patterns of the three models estimated for recording session B4. The horizontal lines indicate approximate boundaries between cortical layers II/III, layer IV and layers V/VI. (**a**) Ising model coupling matrix. Each row/column of the matrix corresponds to a neuron, bias terms are shown on the diagonal. The model has many small coupling terms that encode positive correlations. (**b**) The RBM coupling weights are shown as a bar chart for each hidden unit, ordered from left to right from largest to smallest activity. The first bar chart is the bias for all the visible units, and the blue bar at the top of each plot corresponds to the bias of that hidden unit. Blue bars indicate negative values (the bias terms are predominantly negative, but plotted with flipped sign to fit on the scale of the remaining terms). (**c**) The sRBM weights are shown in the same way, with the pairwise couplings on the left and hidden units on the right. The pairwise connections are qualitatively very different from those of the Ising model, as most of the structure is better captured by hidden units. (**d**) Pairwise coupling terms for the Ising model with stimulus terms. Much of the structure, in particular the bias terms, have been explained away by the stimulus terms. (**e**) Hidden unit couplings for the RBM with stimulus terms. The structure of the hidden units remains largely unchanged, indicating higher-order couplings are due to network interactions and not stimulus correlations.

The estimated model parameters for the three different types of models (Ising, RBM and sRBM) are shown in Fig. 2 for session B4. The 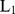 sparseness penalty, chosen to optimize likelihood on a validation dataset, results in many of the parameters being zero. For the Ising model (a) we show the coupling as a matrix plot, with lines indicating anatomical layer boundaries. The diagonal contains the bias terms, which are negative since all cells are off the majority of the time. The matrix has many small positive weights that encourage positive pairwise correlations.

In (b) we show the hidden units of the RBM as individual bar plots, with the bars representing connection strengths to visible units. The topmost bar corresponds to the hidden bias of the unit, and hidden units are ordered from highest to lowest variance. The units are highly selective in connectivity: The first unit almost exclusively connects to cells in the deep (granular and subgranular) cortical layers. The second unit captures correlations between cells in the superficial (supergranular) layers. The correlations are of high order, with 10 and more cells receiving input from a hidden unit. The remaining units connect fewer cells, but still tend to be location-specific. Only the hidden units that have non-zero couplings are shown. Additional hidden units are turned off by the 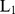 sparseness penalty, which was chosen to maximize likelihood on the cross-validation dataset. The interpretation of hidden units is quite similar to the pairwise terms of the Ising model: positive coupling to a group of visible units encourages these units to become active simultaneously, as the energy of the system is lowered if both the hidden unit and the cells it connects to are active. Thus the hidden units become switched on when cells they connect to are firing (activation of hidden units not shown).

The sRBM combines both pairwise and hidden connections and hence is visualized with a pairwise coupling matrix and bar plots for hidden units. With the larger number of parameters, the best model is even more sparse in the number of nonzero parameters. The remaining pairwise terms predominantly encode negative interactions, and much of the positive coupling has been explained away by the hidden units. These give rise to strong positive couplings within either superficial (II/III) or intermediate (IV) and deep (V/VI) layers, which explain the majority of structure in the data. The more succinct explanation for dependencies between recorded neurons is via connections to shared hidden units, rather than direct couplings between visible units. The RBM and sRBM in this comparison were both estimated with 22 hidden units, but we show only units that did not turn off entirely due to the sparseness penalty. In this example, a sparseness penalty of 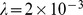 was found to be optimal for all three models.

### Including stimulus-driven components

In order to ascertain to what degree the stimulus driven component of activity accounts for the learned higher-order correlations, we augmented the above models with a dynamic bias term that consists of the log of the average instantaneous firing probability of each cell over repeated presentations of the same stimulus. In the case that all trained parameters were zero, this model would assign a firing probability to all neurons identical to that in the peri-stimulus time histogram (PSTH).

In Fig. 2d) the couplings for the Ising model with stimulus terms are shown. As the pairwise couplings now only capture additional structure beyond correlations explained by the stimulus, they tend to be weaker than in the Ising model without stimulus terms. In particular the bias terms on the diagonal are almost completely explained away by the dynamic bias. The same reasoning applies to the RBM with PSTH terms, which is shown in e). Although the couplings are weaker than for the pure RBM, the basic structure remains, with the first two hidden units explaining correlations within superficial and deep groups of cells, respectively. This shows that the learned coupling structure can not be explained purely from higher-order stimulus correlations and receptive field overlap. Even when stimulus-induced correlations are fully accounted for, the correlation structure captured by the RBM remains similar and higher-order correlations are the dominant driver of correlated firing.

### Model comparison

For a quantitative comparison between models, we computed normalized likelihoods using Annealed Importance Sampling (AIS) to estimate the partition function. For each model, we generated 500 samples through a chain of 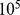 annealing steps. To ensure convergence of the chain, we use a series of chains varying the number of annealing steps and verify that the estimate of the partition function 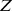 stabilizes to within at least 0.02 bits/s (see Fig. S1). For models of size 20 and smaller we furthermore computed the partition function exactly to verify the AIS estimate.

**Figure 3:**
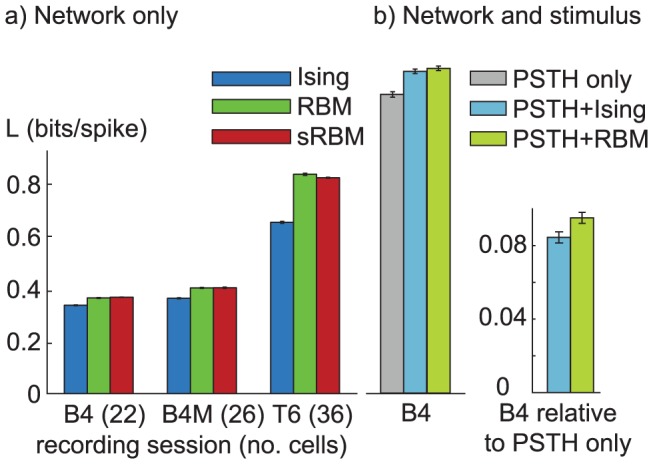
Model comparison using likelihood gain over the independent model. Likelihoods are normalized to bits/spike to account for different population size as well as firing rate. (**a**) The change in performance with dataset size (22 cells for session B4, 26 cells for MU, and 36 cells for T6) is thus due to additional structure captured from larger populations. B4 and T6 are spike sorted, B4M is a multiunit dataset. All three models outperform the independent model by 0.4–0.8 bits/spike. The higher-order models with hidden units give a small (0.03–0.04 bits/spike, about 10%) improvement over the Ising model for the small datasets, growing to 0.18 bits/spike, about 28%, for the dataset with the large population size. (**b**) Including stimulus terms provides a large gain in likelihood, even the stimulus PSTH term alone outperforms the network models by a large margin for this dataset. There is still a significant gain by including coupling terms. The difference between the second order Ising model and higher-order RBM is particularly visible in the right hand plot which shows the gain relative to the PSTH only model. All error bars indicate one standard deviation over repeated estimation on different random subsets of the data for training and validation and random initializations of AIS estimation.

Fig. 3a) shows a comparison of excess log likelihood 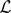 for the three different models and on all three datasets. 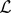, which we define as the gain in likelihood over the independent firing rate model, is computed in units of bits/spike for the full population. Both higher-order models outperform the Ising model in fitting the datasets significantly. Error bars are standard deviation computed from 10 models with different random subsets of the data used for learning and validation, and different random seeds for the parameters and the AIS sampling runs.

Fig. 3b) shows the excess log likelihood for the models with stimulus terms. Due to the additional computational complexity, these models were only estimated for the small B4 data set. The left of the two bar plots shows that including the stimulus information through the PSTH greatly increases the likelihood, even the PSTH only model without coupling terms outperforms the Ising and RBM models by about 0.7 bits/s. Including coupling terms still increases the likelihood, which is particularly visible on the right bar plot which shows the log likelihood gain relative to the PSTH model. Including higher-order coupling terms still provides a significant gain over the pairwise model, confirming that there are higher-order correlation in the data beyond those induced by the stimulus.

Each of the models was estimated for a range of sparseness parameters 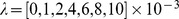 bracketing the optimal 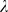 using 4-fold cross-validation on a holdout set, and the results are shown for the optimal choice of 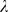 for each model.

**Figure 4:**
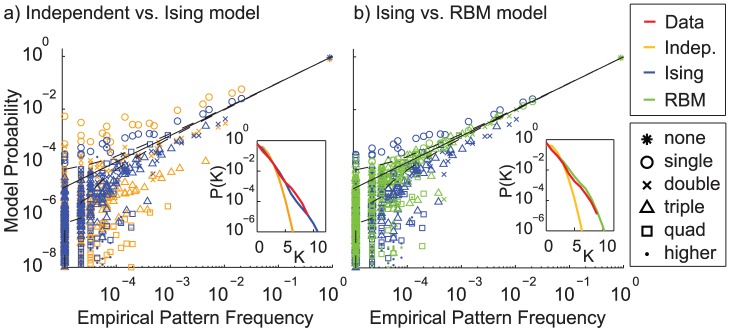
Scatter plot showing empirical probabilities against model probabilities on T6 test dataset. Different models are distinguished by color, the number of simultaneously spiking cells in each pattern by different symbols. (**a**) shows the independent model compared to the Ising model, (**b**) shows the Ising model compared to the RBM. The sRBM is omitted as it is very similar to the RBM. The RBM significantly outperforms simpler models. Inserts in both models show the distribution of synchrony, 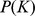, where 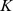 is the number of cells simultaneously firing in one time bin. The synchrony in the empirical distribution (red line) is greatly underestimated by the independent model (yellow), but well captured by both the pairwise (blue) and higher-order model (green).

Additional insight into the relative performance of the models can be gained by comparing model probabilities to empirical probabilities for the various types of patterns. Fig. 4 shows scatter plots of model probabilities under the different models against pattern frequencies in the data. Patterns with a single active cell, two simultaneously active cells, etc. are distinguished by different symbols. As expected from the positive correlations, the independent model (yellow) shown in a) consistently overestimates the probabilities of cells being active individually, so these patterns fall above the identity line, while all other patterns are underestimated. For comparison, the Ising model is shown in the same plot (blue), and does significantly better, indicated by the points moving closer to the identity line. It still tends to fail in a similar way though, with many of the triplet patterns being underestimated as the model cannot capture triplet correlations. In b), this model is directly compared against the RBM (green). Except for very rare patterns, most points are now very close to the identity line, as the model can fully capture higher-order dependencies. Hidden units describe global dependencies that greatly increase the frequency of high order patterns compared to individually active cells. The 5% and 95% confidence intervals for the counting noise expected in the empirical frequency of states are shown as dashed lines. The solid line is the identity. Inserts in both models show the distribution of synchrony, 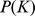, where 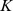 is the number of cells simultaneously firing in one time bin. This metric has been used for example in [18] to show how pairwise models fail to capture higher-order dependencies. In the case of the T6 data set with 36 cells shown here, the Ising model and RBM both provide a good fit to the distribution of synchrony in the observed data.

Note that any error in estimating the partition function of the models would lead to a vertical offset of all points. Thus visually checking the alignment of the data cloud around the identity line provides a visual verification that there are no catastrophic errors in the estimation of the partition function. Unfortunately we cannot use this alignment as a shortcut to compute the partition function without sampling, e.g. by defining 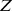 such that the all zeros state has the correct frequency, as this assumes a perfect model fit. For instance, 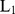 regularization tends to reduce model probabilities of the most frequent states, so this estimate of 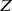 would systematically overestimate the likelihood of regularized models. We note, however, that for higher-order models with no regularization this estimate does indeed agree well with the AIS estimate.

### Spatiotemporal models

The same models can be used to capture spatiotemporal patterns by treating previous time steps as additional cells. Consecutive network states binned at 6.7 ms were concatenated in blocks of up to 13 time steps, for a total network dimensionality of 130 with 10 cells. These models were cross-validated and the sparseness parameters optimized in the same way as for the instantaneous model. This allows us to learn kernels that describe the temporal structure of interactions between cells.

**Figure 5:**
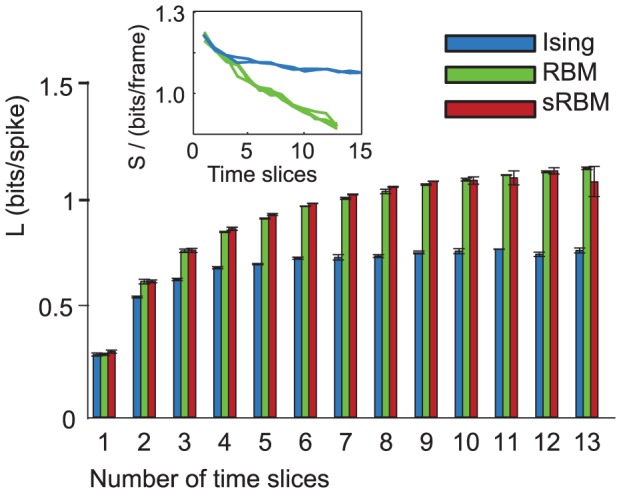
Likelihood comparison as a function of model size. Spatiotemporal models with 10 cells and a varying number of concatenated time steps. The log-likelihood per spike increases as each neuron is modeled as part of a longer time sequence. This effect holds both for Ising and higher-order models. Since the Ising model cannot capture many of the relevant dependencies, the increase in likelihood saturates after about 3 timesteps for the Ising model, but continues to increase for the higher-order models. Insert: Comparison of the entropy per time slice for Ising and RBM models as a function of model size. As the RBM is better able to model spatiotemporal dependencies, the additional entropy for extra frames is smaller than for the Ising models. The RBM does not reach the point of extensivity, where additional frames add constant entropy. Multiple lines of the same color indicate repeated runs with different random initialization.

In Fig. 5 we compare the relative performance of spatiotemporal Ising and higher-order models as a function of the number of time steps included in the model. To create the datasets, we picked a subset of 10 cells with the highest firing rates from the B4 dataset (4 cells from subgranular, 2 from granuar and 4 from supergranular layers) and concatenated blocks of up to 13 subsequent data vectors. This way models of any dimensionality divisible by 10 can be estimated. The number of parameters of the RBM and Ising model were kept the same by fixing the number of hidden units in the RBM to be equal to the number of visible units, the sRBM was also estimated with a square weight matrix for the hidden layer. As before, the higher-order models consistently outperform the Ising model. The likelihood per spike increases with the network size for all models, as additional information from network interactions leads to an improvement in the predictive power of the model. The curve for the Ising model levels off after a dimensionality of about 30 is reached, as higher-order structure that is not well captured by pairwise coupling becomes increasingly important. However, the likelihood of higher-order models continues to increase through the entire experimental range.

The insert in the figure shows the entropy of the models, normalized by the data dimensionality by dividing by the number of frames and neurons. The entropy was computed as 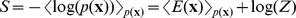 where the expectation of the energy was estimated by initializing 100,000 samples using the holdout data set, and then running 2000 steps of Gibbs sampling. Due to temporal dependencies additional frames carry less entropy, but we do not reach the point of extensivity where the additional entropy per frame reaches a constant value. As the RBM is better able to explain additional structure in new frames, the additional entropy for new frames is much less than for the Ising model.

A similar observation has been made in [5], where Ising and higher-order models for 100 retinal ganglion cells were compared to models for 10 time steps of 10 cells. It is noteworthy that temporal dependencies are similar to dependencies between different cells, in that there are strong higher-order correlations not well described by pairwise couplings. These dependencies extend surprisingly far across time (at least 87 ms, corresponding to the largest models estimated here) and are of such a form that including pairwise couplings to these states does not increase the likelihood of the model. This has implications e.g. for GLMs that are typically estimated with linear spike coupling kernels which will likely miss these interactions.

**Figure 6:**
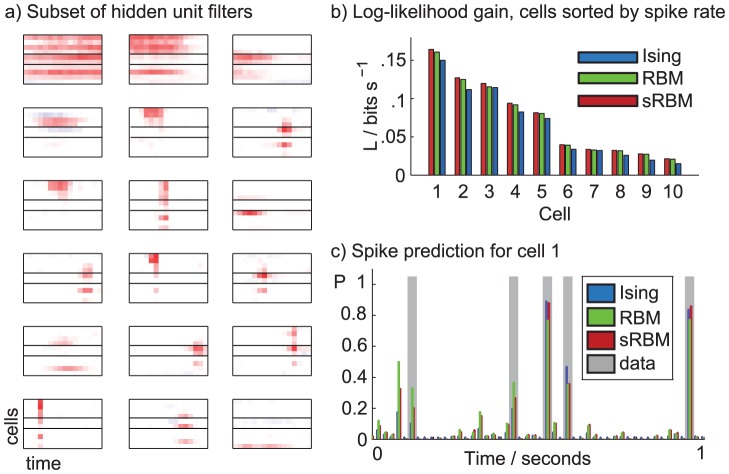
Spatiotemporal network models. (**a**) Hidden units of spatiotemporal sRBM model. For each hidden unit, the horizontal axis is time and the vertical axis cells with horizontal bars separating the subgranular, granular and supergranular cortical layers. (**b**) Log-likelihood gain for each of the 10 cells (ordered by firing rate) conditioned on the remainder of the network state for all three models. (**c**) Spike prediction from network history. For one of the cells, we show 1 s of predicted activity given the history of the network state. In each case when a spike occurs in the data (gray bars), there is an elevated probability under the models (colored bars).

To predict spiking based on the network history, we can compute the conditional distribution of single units given the state of the rest of the network. This is illustrated for a network with 15 time steps for a dimensionality of 150. This model is not included in the above likelihood comparison, as the AIS normalization becomes very expensive for this model size. Fig. 6a) shows the learned weights of 18 randomly chosen nonzero hidden units for a spatiotemporal RBM model with 150 hidden units. Each subplot corresponds to one hidden unit, which connects to 10 neurons (vertical axis) across 15 time steps or 100 ms (horizontal axis). Some units specialize in spatial coupling across different cells at a constant time lag. As the model has no explicit notion of time, the time lag of these spatial couplings is not unique and the model learns multiple copies of the same spatial pattern. Thus while there are 55 nonzero hidden units, the number of unique patterns is much smaller so that the effective representation is quite sparse. The remaining units describe smooth, long-range temporal dependencies, typically for small groups of cells. Both of these subpopulations capture higher-order structure connecting many neurons that cannot be well approximated with pairwise couplings.

By conditioning the probability of one cell at one time bin on the state of the remaining network, we can compute how much information about a cell is captured by the model over a naive prediction based on the firing rate of the cell. This conditional likelihood for each cell is plotted in Fig. 6b) in a similar way to excess log likelihood for the entire population in Fig. 5, except in units of bits per second rather than bits per spike. While the result here reflects our previous observation that Boltzmann machines with hidden units outperform Ising models, we note that the conditional probabilities are easily normalized in closed form since they describe a one-dimensional state space. Thus we can ensure that the likelihood gain holds independent of the estimation of 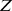 and is not due to systematic errors in sampling from the high-dimensional models. Fig. 6c) provides a more intuitive look at the prediction. For 1 s of data from one cell, where 5 spikes occur, we show the conditional firing probabilities for the three models given 100 ms of history of itself and the other cells. Qualitatively, the models perform well in predicting spiking probabilities, suggesting it might compare favorably to prediction based on GLMs or Ising models [19].

## Discussion

### Contributions

While there has been a resurgence of interest in Ising-like maximum entropy models for describing neural data, progress has been hampered mainly by two problems. First, estimation of energy based models is difficult since these models cannot be normalized in closed form. Evaluating the likelihood of the model thus requires approximations or a numerical integral over the exponential number of states of the model, making maximum likelihood estimation computationally intractable. Even the pairwise Ising model is typically intractable to estimate, and various approximations are required to overcome this problem. Second, the number of model parameters to be estimated grows very rapidly with neural population size. If correlations up to 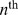 order are considered, the number of parameters is proportional to 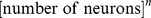. In general, fully describing the distribution over states requires a number of parameters which is exponential in the number of neurons. This can be dealt with by cutting off dependencies at some low order, by estimating only a small number of higher-order coupling terms, or by imposing some specific form on the dependencies.

We attempted to address both of these problems here. Parameter estimation was made tractable using MPF, and latent variables were shown to be an effective way of capturing high order dependencies. This addresses several shortcomings that have been identified with the Ising model.

### Shortcomings of Ising models

As argued in [20], models with direct (pairwise) couplings are not well suited to model data recorded from cortical networks. Since only a tiny fraction of the neurons making up the circuit are recorded, most input is likely to be common input to many of the recorded cells rather than direct synapses between them. While this work compares Generalized Linear Models (GLMs) such as models of the retina [21] and for LGN [22] to linear dynamical systems (LDS) models, the argument applies equally for the models presented here.

Another shortcoming of the Ising model and some previous extensions is that the number of parameters to be estimated does not scale favorably with the dimensionality of the network. The number of pairwise coupling terms in GLM and Ising models scales with the square of the number of neurons, so with the amounts of data typically collected in electrophysiological experiments it is only possible to identify the parameters for small networks with a few tens of cells. This problem is aggravated by including higher-order couplings: for example the number of third order coupling parameters scales with the cube of the data dimensionality. Therefore attempting to estimate these coupling parameters directly is a daunting task that usually requires approximations and strong regularization.

### Higher-order correlations in small networks

Early attempts at modeling higher-order structure side-stepped these technical issues by focussing on structure in very small networks. Ohiorhenuan noted that Ising models fail to explain structure in cat visual cortex [8] and was able to model triplet correlations [9] by considering very small populations of no more than 6 neurons. Similarly, Yu et al. [3], [10] show that over the scale of adjacent cortical columns of anesthetized cat visual cortex, small subnetworks of 10 cells are better characterized with a dichotomized Gaussian model than the pairwise maximum entropy distribution. While the dichotomized Gaussian [23] is estimated only from pairwise statistics, it carries higher-order correlations that can be interpreted as common Gaussian inputs [24]. However these correlations are implicit in the structure of the model and not directly estimated from the data as with the RBM, so it is not clear that the model would perform as well on different datasets.

### Modeling large networks

Given that higher-order correlations are important to include in statistical models of neural activity, the question turns to how these models can be estimated for larger data sets. In this section, we focus on two approaches that are complementary to our model using hidden units. The increasing role of higher-order correlations in larger networks was first observed in [25], where Ising models were fit via MCMC methods to the same 40 cell retina dataset that was analyzed in terms of subsets of 10 cells in [2]. This point is further emphasized by Schneidman and Ganmor in [5], who caution that trying to model small subsets (10 cells) of a larger network to infer properties of the full network may lead to incorrect conclusions, and show that for retinal networks higher-order correlations start to dominate only once a certain network size is reached.

Therefore they address the same question as the present paper, i.e. how to capture 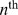 order correlations without the accompanying 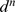 growth in the number of free parameters in a larger network. In their proposed Reliable Interaction Model (RIM), they exploit the sparseness of the neural firing patterns to argue that it is possible to explicitly include third, forth and higher-order terms in the distribution, as most higher-order coupling terms will be zero. Therefore the true distribution can be well approximated from a small number of these terms, which can be calculated using a simple recursive scheme. In practice, the main caveat is that only patterns that appear in the data many times are used to calculate the coupling terms. While the model by construction assigns correct relative probabilities to observed patterns, the probability assigned to unobserved patterns is unconstrained, and most of the RIM's probability mass may thus be assigned to states which never occur in the data.

The second alternative to the RBM with hidden units is to include additional low-dimensional constraints in an Ising model. In the “K-pairwise” model [18], [26], in addition to constraining pairwise correlations, a term is introduced to constrain the probability of 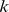 neurons being active simultaneously. This adds very little model complexity, but significantly improves the model of the data. This is shown, for example, by computing conditional predictions in a similar fashion to that shown in Fig. 6c), where the K-pairwise model for a population of 100 retinal ganglion cells has an 80% correlation with the repeated trial PSTH. In contrast to the RBM, however, this model is not structured in a way that can be easily interpreted in terms of functional connectivity. To estimate these models for large numbers (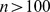) of neurons, the authors leverage the sampling algorithm described in [27], an 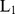-regularized histogram Monte Carlo method.

In addition to proposing a faster (though slower than MPF) parameter estimation method for this class of models, Tkačik and colleagues address the difficulty in sampling from the model and computing the partition function. In our experiments the overall limiting factor is the Gibbs sampler in the AIS partition function estimation. Tkačik et al. use a more efficient sampling algorithm (Wang-Landau) to compute partition functions and entropy of their models. As an even simpler approach to the partition function problem, they suggest that it can be obtained in closed form if the empirical probability of at least one pattern in the data is accurately known. A case in point is the all zeros pattern that is typically frequent for recordings with sparsely firing neurons. Unfortunately, this approach is limited in that it assumes that the probability the model assigns to the state is identical to the empirical probability of the state. In the case that the model has not been perfectly fit to the data, or in the case that the data does not belong to the model class, this will lead to an incorrect estimate of the partition function.

### Modeling stimulus-driven components

Since the activity we are modeling is in response to a specific stimulus, one may rightfully question whether the observed higher-order correlations in neural activity are simply due to higher-order structure contained in the stimulus, as opposed to being an emergent property of cortical networks. In an attempt to tease apart the contribution of the stimulus, we included a nonparametric PSTH term in the model. However, this can capture arbitrarily complex stimulus transformations using the trial-averaged response to predict the response to a new repetition of the same stimulus. As an “oracle model”, it does not only capture the part of the response that could be attributed to a feed-forward receptive field, but also captures contextual modulation effects mediated by surrounding columns and feedback from higher brain areas, essentially making it “too good” as a stimulus model. The RBM and Ising models are then relegated to merely explain the trial to trial variability in our experiments. Not including stimulus terms and finding the best model to explain the correlations present in the data, irrespective of whether they are due to stimulus or correlated variability, seems to be an equally valid approach to discover functional connectivity in the population.

### Relation to GLMs

GLMs [21] can be used to model each cell conditioned on the rest of the population. While mostly used for stimulus response models including stimulus terms, they are easily extended with terms for cross-spike coupling, which capture interactions between cells. GLMs have been successfully augmented with latent variables [20], for instance to model the effect of common noisy inputs on synchronized firing at fast timescales [28]. A major limitation of GLMs is that current implementations can only be estimated efficiently if they are linear in the stimulus features and network coupling terms, so they are not easily generalized to higher-order interactions. Two approaches have been used to overcome this limitation for stimulus terms. The GLM can be extended with additional nonlinearities, preserving convexity on subproblems [22]. Alternatively, the stimulus terms can be packaged into nonlinear features which are computed in preprocessing and usually come with the penalty of a large increase in the dimensionality of the problem [29]. However, we are not aware of any work applying either of these ideas to spike history rather than stimulus terms. Another noteworthy drawback of GLMs is that instantaneous coupling terms cannot be included [20], so instantaneous correlations cannot be modeled and have to be approximated using very fine temporal discretization.

### Conclusion

The RBM provides a parsimonious model for higher-order dependencies in neural population data. Without explicitly enumerating a potentially exponential number of coupling terms or being constrained by only measurements of pairwise correlations, it provides a low-dimensional, physiologically interpretable model that can be easily estimated for populations of 100 or more cells.

The connectivity patterns the RBM learns from cells simultaneously recorded from all cortical layers are spatially localized, showing that small neural assemblies within cortical layers are strongly coupled. This suggests that cells within a layer perform similar computations on common input, while cells across different cortical layers participate in distinct computations and have much less coupled activity. This novel observation is made possible by the RBM: because each of the hidden units responds to (and therefore learns on) a large number of recorded patterns, it can capture dependencies that are too weak to extract with previous models. In particular, the connectivity patterns discovered by the RBM and sRBM are by no means obvious from the covariance of the data or by inspecting the coupling matrix of the Ising model. This approach, combining a straightforward estimation procedure and a powerful model, can be extended from polytrode recordings to capture physiologically meaningful connectivity patterns in other types of multi-electrode data.

## Materials and Methods

### Recording and experimental procedures

#### Ethics statement

The protocol used in the experiments was approved by the Institutional Animal Care and Use Committee at Montana State University and conformed to the guidelines recommended in Preparation and Maintenance of Higher Mammals During Neuroscience Experiments, National Institutes of Health Publication 913207 (National Institutes of Heath, Bethesda, MD 1991).

#### Visual stimuli

Three movies of 8, 20 and 30 minutes duration were captured at 300 frames per second and 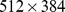 pixel resolution with a Casio F1 camera. All movies were recorded on the campus of MSU Bozeman and depicted natural scenes such as ducks swimming on a pond, selected to contain heterogeneous motion across the scene. The camera was mounted on a tripod to avoid camera motion and there were no scene changes within movies, so the spatiotemporal statistics of the movie do not contain artifacts from camera motion or cuts. Movies were converted to grayscale with no additional contrast normalization on top of the in-camera processing, and temporally down-sampled to 150 Hz for presentation. The high frame rate was chosen to prevent cells phase locking to the frame rate, and scene changes were minimized to avoid evoked potentials due to sudden luminance changes. The movies were presented at 150 frames per second on a 21″ CRT monitor that was calibrated for a linear response. Each of the three movies was presented once and a 30 s segment of the first movie was presented for 60 repeated trials.

#### Recording methods

Data were recorded from anesthetized cat visual cortex in response to a custom set of full field natural movie stimuli. The surgical methods are described in detail elsewhere [30]. In brief, the anesthetized animals were mounted in a stereotaxic frame and a small craniotomy was made over area 17. The dura was reflected and agar in artificial cerebrospinal fluid was applied to protect the cortical surface and reduce pulsations. The polytrode was slowly lowered perpendicularly into cortex using a hydraulic microdrive. Recordings were made with single shank 32 channel polytrodes (Neuronexus Technologies A1×32) with a channel spacing of 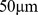, contact diameter of 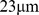 and thickness of 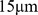, spanning all the layers of visual cortex. Individual datasets had on the order of 20 to 40 simultaneously recorded neurons.

**Figure 7:**
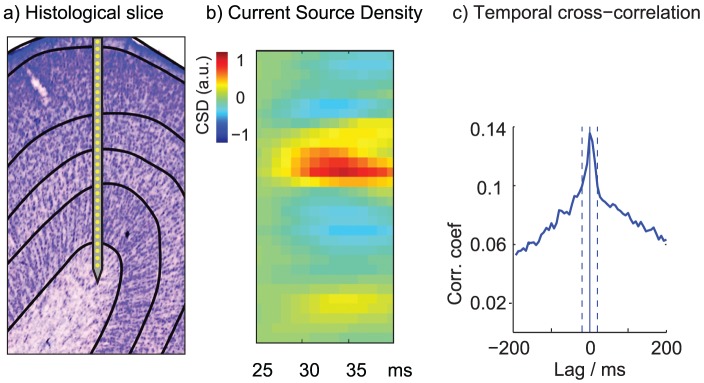
Experimental methods. (**a**) Example recording site with lines indicating layer boundaries and a schematic drawing of the 32-channel probe. (**b**) Current source density (the second spatial derivative of the LFP) in response to a full-field flash stimulus, showing a strong current sink in layer IV and to a lesser degree in layer VI. CSD was used to assign layer boundaries. (**c**) Cross-correlation for one pair of cells across time, binned at 6.7 ms. Correlations fall off quickly as the time lag increases with a central peak extending 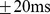 indicated by dashed lines.

For spike sorting, the 32 polytrode channels were treated as 8 non-overlapping groups, shown in Fig. S 2a). To each group, a standard tetrode spike sorting method was applied [30]: In brief, spike waveforms were extracted at a threshold of 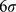 and features (area, energy, peak, valley, peak valley ratio, width, trigger value and the first three principal components) computed for each channel in the group. In this feature space the data was clustered using k-means clustering (KlustaKwik, [31]) with a manual cleanup using the MClust Matlab package [32]. See Fig. S 2b) for cluster isolation for an example channel group. Cleanup consisted of identifying and removing artifacts, and merging clusters belonging to the same cell. Clusters were then labeled as either single- or multi-unit activity based on the following diagnostics: Inspection of distribution of waveform amplitudes on all four channels to ensure clusters were well-separated and no spikes were missing due to the thresholding; inspection of cross-correlograms to identify cells that appeared on two neighboring tetrode groups or triggered on two channels within a group; inspection of inter-spike interval distributions to identify multiunit activity based on intervals shorter than 1 ms and thus below the refractory period of a single cell; inspection of raw waveforms and the time-course of waveform amplitudes. Clusters that passed all criteria were added to the single unit data set, for which ISI histograms are shown in Fig. S 2c), remaining spikes were considered multi-unit activity. Unless noted otherwise, spikes for all data sets were binned at 20 ms where bins with a single spike (2.4% of bins) and multiple spikes (0.9% of bins) were both treated as spiking and the rest as non-spiking. The bin size was chosen to span the width of the central peak in cross-correlograms between pairs of cells such as shown in Fig. 7c), where the dashed vertical lines at 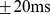 envelope the central peak.

#### Histological procedures

To register individual recording channels with cortical layers, recording locations were reconstructed from Nissl-stained histological sections, and current source density (CSD) analysis in response to 100 repetitions of a full-field stimulus flashed at 1 Hz was used to infer the location of cortical layer IV on the polytrode [33]. Spike-sorted units were assigned to layers based on the channel with the largest amplitude. In Fig. 7a) we use horizontal lines to indicate cortical layer boundaries and their position relative to the polytrode. Fig. 7b) shows the CSD response for this session, showing a strong current sink in layer IV and to a lesser degree in layer VI.

#### Datasets

The models were estimated on a data set of 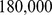 data vectors corresponding to approximately 60 minutes of recording time. The data were split into two subsets of 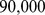 by random assignment of data points: a training set for parameter estimation and a test set to compute cross-validated likelihoods.

For the stimulus dependent model, a separate data set of 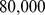 data vectors was used, taken from a 30 s long movie that was repeated for 60 presentations, and again split into a training and test set of equal size. No separate validation set was used and the hyper-parameter was selected directly on the test set.

We also analyzed spatiotemporal patterns of data, which were created by concatenating consecutive state vectors. For the spatiotemporal experiments a bin width of 6.7 ms, corresponding to the frame rate of the stimulus, was used. This bin size is a compromise capturing more detailed structure in the data without leading to an undue increase in dimensionality and complexity. Up to 13 time bins were concatenated in order to discover spatiotemporal patterns and predict spiking given the history over the prior 87 ms. These models were trained on 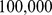 samples and likelihoods were computed on a set of equal size.

### Model and estimation

The sRBM consists of a set of binary visible units 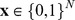 corresponding to observed neurons in the data and a set of hidden units 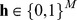 that capture higher-order dependencies. Weights between visible units, corresponding to an Ising model or fully visible Boltzmann machine, capture pairwise couplings in the data. Weights between visible and hidden units, corresponding to an RBM, learn to describe higher-order structure.

The Ising model with visible-visible coupling weights 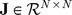 and biases 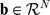 has an energy function
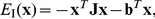
(1)with associated probability distribution 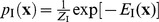, where the normalization constant, or partition function, 
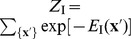
 consists of a sum over all 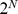 system states.

The RBM with visible-hidden coupling weights 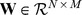 and hidden and visible biases 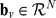 and 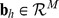 has an energy function
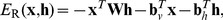
(2)with associated probability distribution 
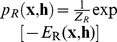
. Since the there are no connections between hidden units (hence “restricted” Boltzmann machine), the latent variables 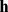 can be analytically marginalized out of the distribution (see supplementary information) to obtain
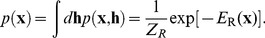
(3)


This step gives a standard energy-based model which we can estimate in our framework, while in a fully connected Boltzmann machine we could not marginalize over hidden units, making the estimation intractable. The energy for the marginalized distribution over 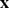 (sometimes referred to as the free energy in machine learning literature) is
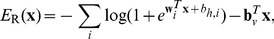
(4)where 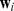 is the 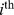 row of the matrix 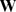. The energy function for an sRBM combines the Ising model and RBM energy terms,
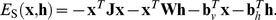
(5)


As with the RBM, it is straightforward to marginalize over the hidden units for an sRBM,
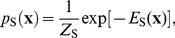
(6)

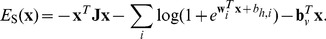
(7)


A hierarchical Markov Random Field based on the sRBM has previously been applied as a model for natural image patches [34], with the parameters estimated using contrastive divergence (CD) [35].

To include stimulus effects into the Boltzmann machine models, we start with a maximum entropy model constrained to fit the peri-stimulus time histogram (PSTH), i.e. the response to a given stimulus obtained by empirically computing the firing probabilities averaged over repeated presentations. This non-parametric model has the form
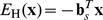
(8)where the subscript 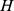 refers to histogram, and both the dynamic bias term 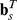 and the data vector 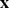 have explicit time dependence. The dynamic bias terms in this model can be computed in closed form 
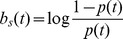
 where the PSTH 
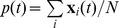
 sums over stimulus repetitions and counts the number of spikes fired by the neuron. Starting with this model and keeping the dynamic bias term fixed at the closed form solution, we add pairwise and higher-order coupling terms to obtain
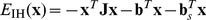
(9)

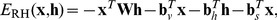
(10)which combine the Ising and RBM population models, respectively, with the stimulus model given by the PSTH term. Estimation of these models is no more difficult than the standard Ising and RBM models, since the PSTH term is computed in closed form and effectively only adds a constant to the energy function. This method corresponds to model T2 (explicit time dependence, second order stimulus dependence) of [36], where it is not further explored due to the requirement for repeated stimulus segments. We used 60 repetitions of a 30 s long natural movie to estimate the PSTH.

Instead of CD, which is based on sampling, we train the models using Minimum Probability Flow (MPF, [13]), a recently developed parameter estimation method for energy based models. MPF works by minimizing the KL divergence between the data distribution and the distribution which results from moving slightly away from the data distribution towards the model distribution. This KL divergence will be uniquely zero in the case where the model distribution is identical to the data distribution. While CD is a stochastic heuristic for parameter update, MPF provides a deterministic and easy to evaluate objective function. Second order gradient methods can therefore be used to speed up optimization considerably. The MPF objective function
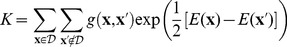
(11)measures the flow of probability out of data states 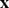 into neighboring non-data states 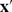, where the connectivity function 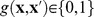 identifies neighboring states, and 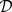 is the list of data states. We consider the case where the connectivity function 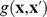 is set to connect all states which differ by a single bit flip
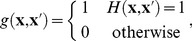
(12)where 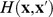 is the Hamming distance between 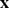 and 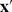. See supplementary information for a derivation of the MPF objective function and gradients for the sRBM, RBM, and Ising models. In all experiments, minimization of K was performed with the MinFunc implementation of L-BFGS [37].

To prevent overfitting all models were estimated with an 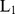 sparseness penalty on the coupling parameters. This was done by adding a term of the form 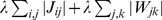 to the objective function, summing over the absolute values of the elements of both the visible and hidden weight matrices. The optimal sparseness 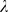 was chosen by cross-validating the log-likelihood on a holdout set, with the value of 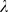 selected from the set 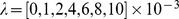 spanning the range of optimal regularization for all models.

Since MPF learning does not give an estimate of the partition function, for models that were too large to normalize by summing over all states, we use annealed importance sampling (AIS, [16]) to compute normalized probabilities. AIS is a sequential Monte Carlo method that works by gradually morphing a distribution with a known normalization constant (in our case a uniform distribution over 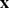) into the distribution of interest. See supplementary information for more detail. AIS applied to RBM models is described in [38], which also highlights the shortcomings of previously used deterministic approximations. Since AIS requires running a sampling chain, in our case a Gibbs sampler, it generally takes much longer than the parameter estimation.

Normalizing the distribution via AIS allows us to compute the log likelihood of the model 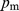 and compare it to the likelihood gained over a baseline model. This baseline assumes cells to be independent and characterized by their firing rate 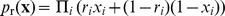 with rates 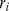 for individual cells 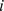. The independent model is easily estimated and normalized, and is commonly used as a reference for model comparison. The excess log likelihood over this baseline is defined in terms of a sample expectation as 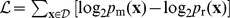.

The excess log likelihood rate 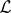, computed in bits/spike by normalizing with the population firing rate per time bin, is used as the basis for model comparisons. Normalizing by the firing rate and comparing bits/spike rather than bits per unit time has the advantage that this measure is less sensitive to the overall activity when comparing across data sets. For models with stimulus terms, a separate normalization constant is required for each state of the dynamic bias. As our 30 s stimulus segment data set consists of 1500 time bins, this amounts to computing 1500 separate partition functions. This limits us to small models with up to 20 neurons where the partition function can be computed quickly by enumerating the full state space, avoiding the use of the costly AIS sampling procedure. To compare likelihoods, we use the PSTH model (i.e. the model with only stimulus and no coupling terms) as the baseline since all three likelihoods are much higher than for models without stimulus terms.

## Supporting Information

Figure S1
**Monitoring the convergence of the AIS estimate for the partition function.** Example shows a 20-dimensional Ising model. Each entry on the horizontal axis corresponds to an annealing chain with a different number of steps. Points correspond to the 500 individual samples, the blue line is the 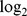 of the average from the samples. The solid green line is the true value of the partition function computed numerically by summing over the 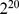 states. The dashed lines correspond to our convergence criterion of 0.02 deviation from the true partition function.(PDF)

Figure S2 **Overview of the data preprocessing and spike sorting procedure (B4 dataset).** Spike waveforms were extracted based on a threshold on each channel and assigned to a unit based on a k-means clustering procedure applied to groups of 4 consecutive channels. (**a**) Mean waveforms of units on 32 channels separated into 8 non-overlapping groups. On each group, the identified units are shown in different colors, and corresponding cell numbers are given. Spikes that could not be assigned to a single unit are captured by MUA clusters. Units 6 and 12 are visibly the same cell picked up on neighboring groups (confirmed by cross-correlation, not shown) and excluded from the data set. (**b**) For single units in group 2, we show the projection of the spikes from 6 different units onto a set of 3 of the total 52 features used for clustering. Similar to spike sorting tetrode data, relative amplitude differences on nearby recording channels provide discrimination between nearby cells. (**c**) Histograms of the inter-spike intervals (ISI) for all 22 single units identified above. The presence of ISIs below the refractory period of a neuron (threshold at 1 ms, shown as a dotted line) provides evidence of multiple neurons being classified in the same cluster. Neurons 17 and 18 have a small amount of contamination with multiunit activity.
(PDF)

Text S1 **Marginalizing over hidden units.** Derivation of the marginal distribution of the RBM so it can be estimated as a standard energy-based model without latent variables.
(PDF)

Text S2 **Objective functions and gradients.** Derivations of the MPF objective function and gradient for the Ising model, RBM and sRBM.
(PDF)

Text S3 **Annealed importance sampling.** Implementation details of the sampling scheme to compute normalized probabilities from the models.
(PDF)
