# Supplementary figures and images for: Modeling Higher-Order Correlations within Cortical Microcolumns
Source: PLoS Comput Biol. 2014 Jul 3;10(7):e1003684. doi: 10.1371/journal.pcbi.1003684 (PMC4081002; doi:10.1371/journal.pcbi.1003684)

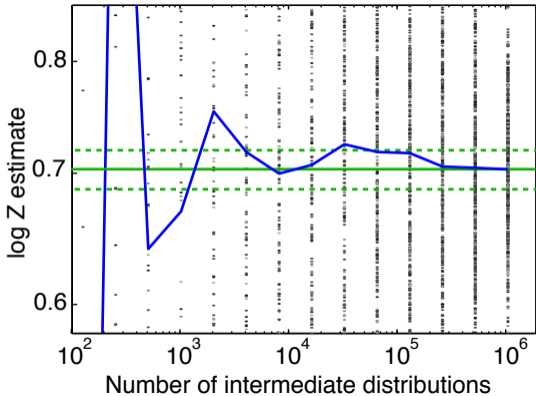

Supplement: Figure S1 — Monitoring the convergence of the AIS estimate for the partition function. Example shows a 20-dimensional Ising model. Each entry on the horizontal axis corresponds to an annealing chain with a different number of steps. Points correspond to the 500 individual samples, the blue line is the of the average from the samples. The solid green line is the true value of the partition function computed numerically by summing over the states. The dashed lines correspond to our convergence criterion of 0.02 deviation from the true partition function. (PDF) [file pcbi.1003684.s001.pdf]
