# Supplementary material for: Modeling Higher-Order Correlations within Cortical Microcolumns
Source: PLoS Comput Biol. 2014 Jul 3;10(7):e1003684. doi: 10.1371/journal.pcbi.1003684 (PMC4081002; doi:10.1371/journal.pcbi.1003684)
Supplement: Figure S2 — Overview of the data preprocessing and spike sorting procedure (B4 dataset). Spike waveforms were extracted based on a threshold on each channel and assigned to a unit based on a k-means clustering procedure applied to groups of 4 consecutive channels. (a) Mean waveforms of units on 32 channels separated into 8 non-overlapping groups. On each group, the identified units are shown in different colors, and corresponding cell numbers are given. Spikes that could not be assigned to a single unit are captured by MUA clusters. Units 6 and 12 are visibly the same cell picked up on neighboring groups (confirmed by cross-correlation, not shown) and excluded from the data set. (b) For single units in group 2, we show the projection of the spikes from 6 different units onto a set of 3 of the total 52 features used for clustering. Similar to spike sorting tetrode data, relative amplitude differences on nearby recording channels provide discrimination between nearby cells. (c) Histograms of the inter-spike intervals (ISI) for all 22 single units identified above. The presence of ISIs below the refractory period of a neuron (threshold at 1 ms, shown as a dotted line) provides evidence of multiple neurons being classified in the same cluster. Neurons 17 and 18 have a small amount of contamination with multiunit activity. (PDF) [file pcbi.1003684.s002.pdf]

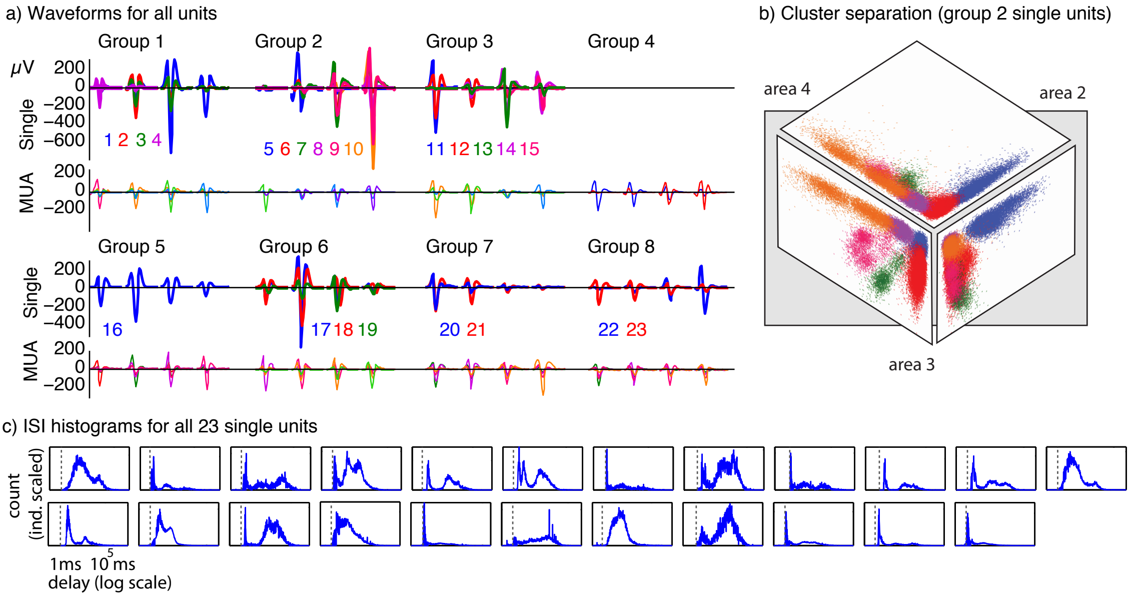

a) Waveforms for all units
Group 1
Group 2
Group 3
Group 4
μV
200
0
−200
−400
−600
Single
1 2 3 4
5 6 7 8 9 10
11 12 13 14 15
200
0
−200
MUA
Group 5
Group 6
Group 7
Group 8
200
0
−200
−400
Single
16
17 18 19
20 21
22 23
200
0
−200
MUA
b) Cluster separation (group 2 single units)
area 4
area 2
area 3
c) ISI histograms for all 23 single units
count (ind. scaled)
1ms 10^5 ms
delay (log scale)
