# Supplementary material for: Modeling Higher-Order Correlations within Cortical Microcolumns
Source: PLoS Comput Biol. 2014 Jul 3;10(7):e1003684. doi: 10.1371/journal.pcbi.1003684 (PMC4081002; doi:10.1371/journal.pcbi.1003684)
Supplement: Text S1 — Marginalizing over hidden units. Derivation of the marginal distribution of the RBM so it can be estimated as a standard energy-based model without latent variables. (PDF) [file pcbi.1003684.s003.pdf]

Text S1

Marginalizing over hidden units

In this section we review how the joint probability for visible and hidden units in the RBM can be integrated in closed form to obtain the marginal distribution for the visible units. First we rewrite the sum over hidden variables as a product

$$p(\mathbf{x},\mathbf{h}) = \frac{1}{Z}\exp\left(\sum_{i,j} x_i h_j w_{ij} + \sum_i x_i a_i + \sum_j h_j b_j\right) \tag{1}$$

$$= \frac{1}{Z} e^{\sum_i x_i a_i} \prod_{j=1}^{M} \exp\left(\sum_i x_i h_j w_{ij} + h_j b_j\right) \tag{2}$$

and integrate $p(\mathbf{x}) = \sum_{\{h\}} p(\mathbf{x},\mathbf{h})$, where the sum is over all configurations of the hidden units. For the purpose of this derivation, we move the first term outside the product

$$p(\mathbf{x}) = \frac{1}{Z} e^{\sum_i x_i a_i} \sum_{\{h\}} \exp\left(\sum_i x_i h_1 w_{i1} + h_1 b_1\right) \prod_{j=2}^{M} \exp\left(\sum_i x_i h_j w_{ij} + h_j b_j\right). \tag{3}$$

We can write the sum over all hidden states as nested sums over every state for each hidden unit

$$p(\mathbf{x}) = \frac{1}{Z} e^{\sum_i x_i a_i} \sum_{h_1\in\{0,1\}} \ldots \sum_{h_m\in\{0,1\}} \exp\left(\sum_i x_i h_1 w_{i1} + h_1 b_1\right) \tag{4}$$

$$\prod_{j=2}^{M} \exp\left(\sum_i x_i h_j w_{ij} + h_j b_j\right). \tag{5}$$

Noting that the term we have singled out appears only in one of the sums, we rearrange to isolate the sum,

$$p(\mathbf{x}) = \frac{1}{Z} e^{\sum_i x_i a_i} \sum_{h_1\in\{0,1\}} \exp\left(\sum_i x_i h_1 w_{i1} + h_1 b_1\right) \tag{6}$$

$$\sum_{h_2\in\{0,1\}} \ldots \sum_{h_m\in\{0,1\}} \prod_{j=2}^{M} \exp\left(\sum_i x_i h_j w_{ij} + h_j b_j\right). \tag{7}$$

Doing this for all terms we obtain a product over individual one-dimensional sums

$$p(\mathbf{x}) = \frac{1}{Z} e^{\sum_i x_i a_i} \prod_{j=1}^{M} \sum_{h_j\in\{0,1\}} \exp\left(\sum_i x_i h_j w_{ij} + h_j b_j\right) \tag{8}$$

$$= \frac{1}{Z} e^{\sum_i x_i a_i} \prod_{j=1}^{M} \left[1 + \exp\left(\sum_i x_i w_{ij} + b_j\right)\right] \tag{9}$$

$$= \frac{1}{Z} \exp\left(\sum_i x_i a_i \sum_{j=1}^{M} \log\left[1 + \exp\left(\sum_i x_i w_{ij} + b_j\right)\right]\right). \tag{10}$$

The marginal distribution over $\mathbf{x}$ for the RBM is now in the form of a standard energy based model, with energy function

$$E(\mathbf{x}) = -\sum_i x_i a_i - \sum_{j=1}^{M} \log\left[1 + \exp\left(\sum_i x_i w_{ij} + b_j\right)\right]. \tag{11}$$

The sRBM follows the same logic, since additional terms in the energy that do not depend on the hidden units stay outside the sum over hidden configurations in the same fashion as for the visible bias.
