# Supplementary material for: Modeling Higher-Order Correlations within Cortical Microcolumns
Source: PLoS Comput Biol. 2014 Jul 3;10(7):e1003684. doi: 10.1371/journal.pcbi.1003684 (PMC4081002; doi:10.1371/journal.pcbi.1003684)
Supplement: Text S2 — Objective functions and gradients. Derivations of the MPF objective function and gradient for the Ising model, RBM and sRBM. (PDF) [file pcbi.1003684.s004.pdf]

Text S2

MPF objective

Ising model

In this section we review the derivation of the MPF objective for an Ising model, where the objective function consists of terms connecting the data states to all states which differ by a single bit flip. The general MPF objective function is given by

$$K(\boldsymbol{\Theta}) = \sum_{\mathbf{x}\in\mathcal{D}} \sum_{\mathbf{x}'\notin\mathcal{D}} g(\mathbf{x},\mathbf{x}') \exp\left(\frac{1}{2}\left[E(\mathbf{x};\boldsymbol{\Theta}) - E(\mathbf{x}';\boldsymbol{\Theta})\right]\right), \tag{1}$$

where $g(\mathbf{x},\mathbf{x}') = g(\mathbf{x}',\mathbf{x}) \in \{0,1\}$ is the connectivity function, $E(\mathbf{x};\boldsymbol{\Theta})$ is an energy function parameterized by $\boldsymbol{\Theta}$, and $\mathcal{D}$ is the list of data states. We consider the case where the connectivity function $g(\mathbf{x},\mathbf{x}')$ is set to connect all states which differ by a single bit flip,

$$g(\mathbf{x},\mathbf{x}') = \begin{cases} 1 & \mathbf{x} \text{ and } \mathbf{x}' \text{ differ by a single bit flip}, \quad \sum_n |x_n - x'_n| = 1 \\ 0 & \text{otherwise} \end{cases} \tag{2}$$

The MPF objective function in this case is

$$K(\boldsymbol{\Theta}) = \sum_{\mathbf{x}\in\mathcal{D}} \sum_{n=1}^{N} \exp\left(\frac{1}{2}\left[E(\mathbf{x};\boldsymbol{\Theta}) - E(\mathbf{x}+\mathbf{d}(\mathbf{x},n);\boldsymbol{\Theta})\right]\right) \tag{3}$$

where the sum over n is a sum over all data dimensions, and the bit flipping function $\mathbf{d}(\mathbf{x},n) \in \{-1,0,1\}^N$ is

$$\mathbf{d}(\mathbf{x},n)_i = \begin{cases} 0 & i \neq n \\ -(2x_i - 1) & i = n \end{cases} \tag{4}$$

For the Ising model, the energy function is

$$E = \mathbf{x}^T \mathbf{J} \mathbf{x} \tag{5}$$

where $\mathbf{x} \in \{0,1\}^N$, $\mathbf{J} \in \mathcal{R}^{N\times N}$, and $\mathbf{J}$ is symmetric ($\mathbf{J} = \mathbf{J}^T$). The bias terms have been absorbed into the diagonal of the matrix $\mathbf{J}$ which is possible since $x^2 = x$ holds for binary $\mathbf{x}$.

Substituting this energy into the MPF objective function, it becomes

$$K = \sum_{\mathbf{x}\in\mathcal{D}} \sum_{n} \exp\left(\frac{1}{2}\left[\mathbf{x}^T\mathbf{J}\mathbf{x} - (\mathbf{x}+\mathbf{d}(\mathbf{x},n))^T\mathbf{J}(\mathbf{x}+\mathbf{d}(\mathbf{x},n))\right]\right) \tag{6}$$

$$= \sum_{\mathbf{x}\in\mathcal{D}} \sum_{n} \exp\left(\frac{1}{2}\left[\mathbf{x}^T\mathbf{J}\mathbf{x} - \left(\mathbf{x}^T\mathbf{J}\mathbf{x} + 2\mathbf{x}^T\mathbf{J}\mathbf{d}(\mathbf{x},n) + \mathbf{d}(\mathbf{x},n)^T\mathbf{J}\mathbf{d}(\mathbf{x},n)\right)\right]\right) \tag{7}$$

$$= \sum_{\mathbf{x}\in\mathcal{D}} \sum_{n} \exp\left(-\frac{1}{2}\left[2\mathbf{x}^T\mathbf{J}\mathbf{d}(\mathbf{x},n) + \mathbf{d}(\mathbf{x},n)^T\mathbf{J}\mathbf{d}(\mathbf{x},n)\right]\right) \tag{8}$$

$$= \sum_{\mathbf{x}\in\mathcal{D}} \sum_{n} \exp\left(-\frac{1}{2}\left[2\sum_i x_i J_{in}(1-2x_n) + J_{nn}\right]\right) \tag{9}$$

$$= \sum_{\mathbf{x}\in\mathcal{D}} \sum_{n} \exp\left(\left[(2x_n - 1)\sum_i x_i J_{in} - \frac{1}{2}J_{nn}\right]\right). \tag{10}$$

Assume the symmetry constraint on $\mathbf{J}$ is enforced by writing it in terms of a another possibly asymmetric matrix $\mathbf{J}' \in \mathcal{R}^{N\times N}$,

$$\mathbf{J} = \frac{1}{2}\mathbf{J}' + \frac{1}{2}\mathbf{J}'^T. \tag{11}$$

The derivative of the MPF objective function with respect to $\mathbf{J}'$ is

$$\begin{aligned}\frac{\partial K}{\partial J'_{lm}} = &\frac{1}{2}\sum_{\mathbf{x}\in\mathcal{D}} \exp\left(\left[(2x_m-1)\sum_i x_i J_{im} - \frac{1}{2}J_{mm}\right]\right)\left[(2x_m-1)\,x_l - \delta_{lm}\frac{1}{2}\right] \\ &+ \frac{1}{2}\sum_{\mathbf{x}\in\mathcal{D}} \exp\left(\left[(2x_l-1)\sum_i x_i J_{il} - \frac{1}{2}J_{ll}\right]\right)\left[(2x_l-1)\,x_m - \delta_{ml}\frac{1}{2}\right],\end{aligned} \tag{12}$$

where the second term is simply the first term with indices l and m reversed.

RBM

After marginalizing out the hidden units, the energy function over the visible units for an RBM is given by:

$$E(\mathbf{x}) = -\sum_i \log(1 + e^{-W_i\mathbf{x}}) \tag{13}$$

where W_i is a vector of coupling parameters and $\mathbf{x}$ is the binary input vector. Bias terms have been omitted for readability.

As previously, we substitute into the objective function Eq. 3 to obtain

$$K = \sum_{\mathbf{x}\in\mathcal{D}}\sum_n \exp\left(\frac{1}{2}\left[-\sum_i \log\left(1+e^{-W_i\mathbf{x}}\right) + \sum_i \log\left(1+e^{-W_i\mathbf{x}+W_i\mathbf{d}(\mathbf{x},n)}\right)\right]\right). \tag{14}$$

Unlike for the Ising model there is no cancellation of data and non-data energy terms, so evaluating the function and derivative requires looping over all bit flips for the data set.

sRBM

The energy function over the visible units for an sRBM obtained by marginalizing out the conditionally independent hidden units is

$$E(\mathbf{x};\mathbf{J},\mathbf{W}) = \mathbf{x}^T\mathbf{J}\mathbf{x} - \sum_i \log(1+e^{-\mathbf{W}_i^T\mathbf{x}}) \tag{15}$$

where $\mathbf{x} \in \{0,1\}^N$ is the visible state, $\mathbf{J} = \mathbf{J}^T \in \mathcal{R}^{N\times N}$ is a symmetric coupling matrix, and $\mathbf{W} \in \mathcal{R}^{M\times N}$ is a weight matrix to M hidden units. Equation 15 consists of a term capturing connections between visible units (an Ising model), and a term capturing connections to hidden units (an RBM).

The MPF objective we use again consists of energy differences between data and non-data states differing by a single bit. For the RBM this energy difference with the nth bit flipped is

$$\begin{aligned} dE_n^{\mathrm{R}} &= -\sum_i \left[\log(1+e^{-\mathbf{w}_i^T\mathbf{x}}) - \log(1+e^{-\mathbf{w}_i^T(\mathbf{x}+d(\mathbf{x},n))})\right] \qquad (16)\\ &= -\sum_i \left[\log(1+e^{z_i}) - \log(e^{z_i}+e^{w_{in}b_n})\right] \qquad (17)\end{aligned}$$

where for notational simplicity we have defined $z_i = \mathbf{w}_i^T \mathbf{x}$ and $b = 2\mathbf{x} - 1$. The energy difference contributed by connections between visible units (the Ising model) is

$$dE_n^{\mathrm{I}} = 2b_n y_n - \frac{1}{2} J_{nn} \tag{18}$$

where we define the shorthand $\mathbf{y} = \mathbf{J}\mathbf{x}$ for simplicity. The total objective function is then given by a sum over samples and bit flips as

$$K = \sum_{\mathbf{x} \in \mathcal{D}} \sum_n \exp\left[\frac{1}{2}(dE_n^{\mathrm{I}} + dE_n^{\mathrm{R}})\right] \tag{19}$$

To compute the gradient of this objective w.r.t. the parameters W and J we not that

$$\frac{\partial K}{\partial J} = \sum_{\mathbf{x} \in \mathcal{D}} \sum_n K_n \frac{\partial}{\partial J} dE_n^{\mathrm{I}} \tag{20}$$

$$\frac{\partial K}{\partial W} = \sum_{\mathbf{x} \in \mathcal{D}} \sum_n K_n \frac{\partial}{\partial W} dE_n^{\mathrm{R}} \tag{21}$$

these terms are computed as

$$\frac{\partial}{\partial J} dE_n^{\mathrm{I}} = \frac{\partial}{\partial J}\left(2b_n y_n - \frac{1}{2} J_{nn}\right) = 2b_n x_n - \frac{1}{2} \tag{22}$$

for the pairwise terms, and

$$\frac{\partial}{\partial W_{ab}} dE_n^{\mathrm{R}} = -\frac{\partial}{\partial W_{ab}} \sum_i \left[\log(1 + e^{z_i}) - \log(e^{z_i} + e^{w_{in} b_n})\right] \tag{23}$$

$$= \frac{1}{2} \sum_n \frac{e^{z_a}}{1 + e^{z_a}} x_b \tag{24}$$

$$+ \frac{1}{2} \sum_n \frac{e^{z_a}}{e^{z_a} + e^{w_{an} b_n}} x_b \tag{25}$$

$$+ \frac{1}{2} \sum_j \frac{1}{1 + e^{z_a - w_{ab} b_b}} b_b \tag{26}$$

for the higher order terms.
