# Supplementary material for: Modeling Higher-Order Correlations within Cortical Microcolumns
Source: PLoS Comput Biol. 2014 Jul 3;10(7):e1003684. doi: 10.1371/journal.pcbi.1003684 (PMC4081002; doi:10.1371/journal.pcbi.1003684)
Supplement: Text S3 — Annealed importance sampling. Implementation details of the sampling scheme to compute normalized probabilities from the models. (PDF) [file pcbi.1003684.s005.pdf]

Text S3

Annealed importance sampling

Estimating the normalization constant, also referred to as partition function, of an energy based probabilistic model, remains a challenging task [1]. Many approaches to make learning the parameters of energy based models tractable, such as Contrastive Divergence, MPF, Score and Ratio Matching [2, 3] do not attempt to estimate the partition function. A notable exception is Noise Contrastive Estimation [4] which treats the partition function as a parameter to be estimated, but has only been applied for continuous-valued data. Most commonly the partition function is estimated by sampling.

Using importance sampling, the partition function can be estimated by

$$Z_p/Z_q = \left\langle \frac{\tilde{p}(\mathbf{x})}{\tilde{q}(\mathbf{x})} \right\rangle_{q(\mathbf{x})} \tag{1}$$

where Z_q is the known partition function of the proposal distribution $q(\mathbf{x})$, Z_p is the partition function of interest for $p(\mathbf{x})$ and the tilde symbol indicates a non-normalized distribution. The angle brackets indicate a sample expectation over samples from the distribution $p(\mathbf{x})$. However, if $q(\mathbf{x})$ is not a good match to the target distribution, it takes a very large number of samples to get a good estimate. AIS uses an annealing process to gradually transform a simple proposal distribution, such as the uniform distribution, into the target distribution, leading to an accurate estimate of Z from only a small number of samples.

To assure convergence of the estimator, we run several annealing chains, increasing the number of steps in factors of 2 up to a size of 10^5 steps. We check that the final estimate of $\log_2(Z)$ does not deviate more than 0.02 from the previous estimates. This criterium was chosen since $\log_2(Z)$ appears as an additive term to $\mathcal{L}$, and at a bin size $\tau = 50$ms an error of 1 bits / second in the final estimate of the likelihood was seen as an acceptable trade-off between estimation speed and accuracy. In Fig. S1, we show this convergence plot for a small 20-dimensional model, where the normalization constant was computed exactly. For larger models, where the partition function could not be calculated analytically, we monitored that the estimate stabilized to within this tolerance.

References

1. Salakhutdinov R, Murray I (2008) On the quantitative analysis of deep belief networks. In: ICML. URL `http://dl.acm.org/citation.cfm?id=1390266`.

2. Hyvärinen A (2006) Estimation of non-normalized statistical models by score matching. Journal of Machine Learning Research 6: 695–709.

3. Hyvärinen A (2007) Some extensions of score matching. Computational statistics & data analysis 51.

4. Gutmann M (2009) Noise-contrastive estimation : A new estimation principle for unnormalized statistical models. Journal of Machine Learning Research : 297–304.
